# Strategies to reduce osmotic stress during cryopreservation of red blood cells when using trehalose

**DOI:** 10.1038/s41598-026-61563-x

**Published:** 2026-07-09

**Authors:** Tobias Braun, Harriëtte Oldenhof, Inola-Trinity Kohrs, Rainer Blasczyk, Constança Figueiredo, Willem F. Wolkers

**Affiliations:** 1 Biostabilization laboratory - Lower Saxony Centre for Biomedical Engineering, Implant Research and Development, University of Veterinary Medicine Hannover, Hannover, Germany; 2 Unit for Reproductive Medicine - Clinic for Horses, University of Veterinary Medicine Hannover, Hannover, Germany; 3 Institute of Transfusion Medicine and Transplant Engineering, Hannover Medical School, Hannover, Germany

**Keywords:** Betaine, Cryopreservation, Erythrocytes, Freeze-drying, Membrane permeability, Trehalose loading, Biochemistry, Biotechnology

## Abstract

The aim of this study was to determine optimal conditions for cryopreserving red blood cells (RBCs) using trehalose. We assessed the extent of trehalose uptake by RBCs during incubation at 37 °C and following freezing-and-thawing. Additionally, we examined whether betaine could alleviate osmotic stress at various stages of the cryopreservation process, including trehalose loading, freezing, and return to isotonic conditions after thawing. Trehalose uptake during incubation at 37 °C was limited, whereas freezing-and-thawing RBCs in trehalose-containing solutions led to substantially higher intracellular trehalose concentrations. The greatest post-thaw survival was observed with 400 mM trehalose in combination with rapid cooling. When trehalose was combined with betaine, optimal cryosurvival was maintained at a total solute concentration of 400 mM, whereas supplementing 400 mM trehalose with membrane-permeating cryoprotectants such as glycerol or dimethyl sulfoxide (DMSO) further enhanced cell cryosurvival. Membrane permeability studies indicated that betaine acts as a non-permeating solute contradicting literature findings. Direct transfer of trehalose-loaded, cryopreserved RBCs to isotonic conditions after thawing resulted in hemolysis, but this could be reduced by using hypertonic washing solutions. In conclusion, no synergistic protective effect was observed from combining betaine with trehalose for RBC cryopreservation, whereas combinations of trehalose with membrane-permeating agents like glycerol or DMSO appear promising for improving RBC cryopreservation outcomes.

## Introduction

Red blood cell (RBC) transfusions are essential for supporting surgical procedures and treating hematological disorders. Blood banks supply packed RBC concentrates that are stored at 4 °C under hypothermic conditions for clinical use^1^. However, the quality of these concentrates progressively deteriorates during storage, compromising their therapeutic effectiveness^2–5^. Alternatively, for long-term preservation, blood units may be cryopreserved. Trehalose has attracted considerable attention as a cryoprotective agent (CPA) for RBCs because of its low toxicity and suitability for freeze-drying applications^6–8^. Despite its effectiveness as cryoprotective agent, exposure of cells to trehalose during loading and freezing, and trehalose removal after thawing cause osmotic imbalances that result in hemolysis. This occurs because trehalose does not readily cross cellular membranes^6,9^.

Glycerol, a membrane-permeating agent, is the most commonly used CPA for RBC cryopreservation^1^. However, this approach is not routinely applied in clinics because it requires relatively high glycerol concentrations (20–40%) and labor-intensive loading and removal steps to minimize post-thaw cell losses and adverse side effects from residual glycerol^10,11^. Trehalose is an attractive alternative CPA due to its biocompatibility and the fact that it does not need to be removed prior to transfusion. To address the limited permeability of cell membranes to trehalose, various strategies have been explored, including electroporation^12^, sonoporation^13^, genetic modification^14^, and the use of membrane-permeabilizing agents^15,16^. Additionally, membrane phase transitions induced by heating^17,18^ or cooling^19,20^ can temporarily increase membrane permeability, enabling trehalose transport across membranes.

Although several of the aforementioned loading strategies enable efficient trehalose uptake, intracellular accumulation of trehalose generates a hypertonic intracellular environment relative to the extracellular milieu when cells are returned to isotonic conditions. This osmotic imbalance when trehalose-loaded RBCs are returned to isotonic conditions causes hemolysis, which continues to pose a significant challenge in the practical use of trehalose for RBC cryopreservation^21^. In nature, certain cells accumulate osmolytes, small organic molecules, that help restore osmotic balance. Osmolytes such as betaine, proline, ectoine, and glycerol can traverse cell membranes via specific transporters^22^. It has therefore been proposed that supplementing disaccharide-based freezing formulations with such osmolytes may improve cryopreservation outcomes for RBCs and other cell types^21,23–25^. In this study, we tested if betaine can act as a membrane permeating agent to alleviate hypertonic and hypotonic stresses associated with cryopreservation of red blood cells with trehalose as previously suggested^21^, and corroborated this with studies on the permeability of human red blood cell membranes to betaine.

Cell viability following cryopreservation depends not only on the type and contents of the CPAs that are used, but also on the cooling rate applied. Excessively slow cooling leads to solution effects injury, whereas too rapid cooling can cause lethal intracellular ice formation. Consequently, cells display a distinct optimal cooling rate at which post-thaw survival is maximized^26–28^. For human RBCs cryopreserved with 40% glycerol, the optimal cooling rate ranges from 1 to 3 °C min⁻¹, while use of lower glycerol concentrations (20%) require substantially higher cooling rates (> 60 °C min⁻¹) to achieve maximal survival^10,11^. Trehalose-based cryopreservation approaches for RBCs typically require rapid cooling, such as direct immersion of 1-mL samples into liquid nitrogen, for good cryopreservation outcome^29–31^.

In this study, we aimed to establish optimal conditions for trehalose-based cryopreservation of RBCs, with particular emphasis on minimizing hypertonic and hypotonic stresses during trehalose loading, freezing, and returning cells to isotonic conditions after thawing. Specifically, we (1) evaluated the efficiency of trehalose loading during incubation at 37 °C and following freeze-thaw exposure; (2) determined optimal cooling rates for cryopreservation; (3) investigated whether supplementation with the osmolyte betaine enhances trehalose uptake and post-thaw survival by modulating osmotic responses; (4) assessed membrane permeability to various CPAs; and (5) examined whether wash media supplemented with betaine or permeating agents such as glycerol can reduce hemolysis upon returning trehalose-loaded RBCs to isotonic conditions.

## Results

### Loading of red blood cells with trehalose, through incubation at 37 °C

**Fig. 1:**
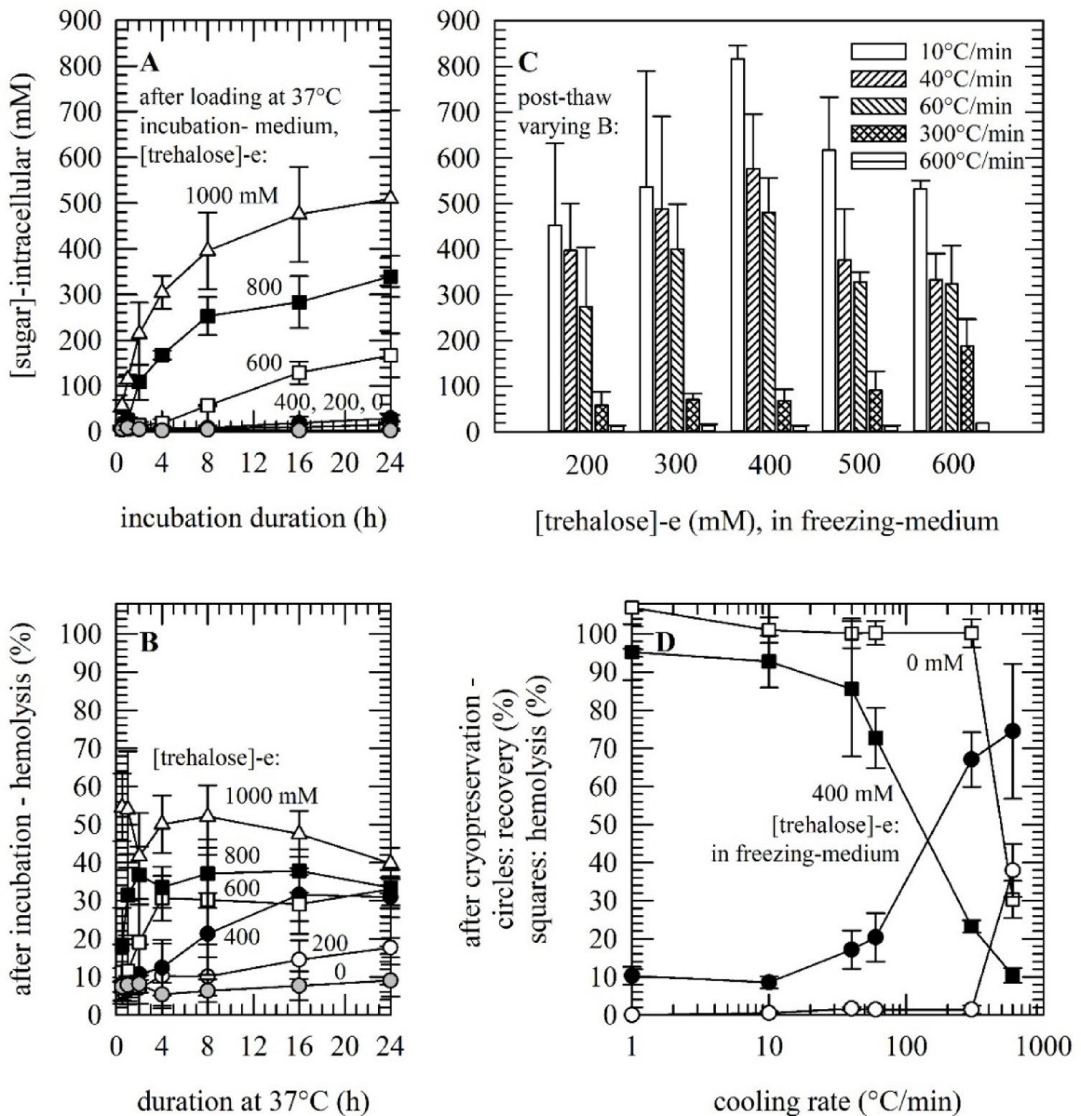
Intracellular sugar concentrations and red blood cell survival after incubation at 37 °C for different durations **(A**,** B)**, or after subjecting to cryopreservation with different cooling rates **(C**,** D)**. RBCs were suspended in 1×PBS supplemented with 0 − 1000 mM trehalose. Incubations at 37 °C were done up to 24 h, whereas cryopreservation was performed with cooling rates ranging from 1–600 °C min^− 1^. In case of incubation at 37 °C, intracellular sugar concentrations **(A)** and hemolysis **(B)** are plotted versus the incubation duration (grey circles: no trehalose; white circles: 200, black circles: 400, open squares: 600, closed squares: 800, open triangles: 1000 mM trehalose). Intracellular sugar concentrations determined after cryopreservation are presented versus the extracellular trehalose concentration (**C**; bars without markings: 10, upwards diagonals: 40, downwards diagonals: 60, crossed: ~300, and horizontal lines: ~600 °C min^− 1^), while post-thaw hemolysis (circles) and recovery (squares) are presented versus the cooling rates used (**D**; white symbols: PBS without trehalose, black symbols: with added 400 mM extracellular trehalose). Values are presented as average ± standard deviation, determined following a split sample approach, using RBC samples originating from three **(A, B)** and four **(C, D)** different donors.

Figure 1A shows that RBCs take up trehalose during incubation at 37 °C in medium supplemented with trehalose, in a time and concentration dependent manner. Intracellular trehalose concentrations of 396 ± 84 mM were found after 8 h with 1 M extracellular trehalose, increasing up to 510 ± 193 mM after 24 h. After 8 h incubation with 800 and 600 mM extracellular trehalose, intracellular sugar concentrations were found to be 253 ± 41 and 57 ± 2 mM, respectively. Prolonged incubations with high extracellular sugar concentrations, however, coincide with high hemolysis levels (> 30%). Hemolysis levels remain below 10% in case of 8 h exposure with up to 400 mM extracellular trehalose. (Fig. 1B) In this case, however, intracellular trehalose contents are less than 10 mM. Sucrose displays similar kinetics for sugar uptake and concomitant hemolysis levels compared to trehalose (data not shown). We note that trehalose uptake and concomitant hemolysis are the result of membrane damage resulting from prolonged hypertonic stress.

### Freezing induced trehalose-uptake by red blood cells, at different cooling rates

Freezing of RBCs in medium supplemented with trehalose results in a cooling rate dependent sugar uptake (Fig. 1C). Freezing-induced trehalose uptake decreases with increasing cooling rate. Also, post-thaw hemolysis and the amount of recovered intact cells are dependent on the cooling rate (Fig. 1D). When cooling rates ranging between 1 and 60 °C min^− 1^ were used, intracellular trehalose concentrations ranged from 480 ± 76 to 816 ± 29 mM, with an optimum at 400 mM extracellular trehalose (Fig. 1C). For some conditions, e.g., when cell recoveries were low (< 20%), intracellular concentrations displayed loading efficiencies greater than 100%, likely because low cell recoveries for those conditions cause inaccuracies. Use of fast cooling rates drastically increases cell recovery and decreases hemolysis. Cooling at ~ 300 °C min^− 1^, with 400 mM extracellular trehalose, resulted in a 67 ± 7% cell recovery, 23 ± 2% hemolysis, and a 68 ± 25 mM intracellular trehalose concentration. Interestingly, in the absence of trehalose, where cell cryosurvival is negligible up to cooling rates of ~ 300 °C min^− 1^ (plunging samples in vials into liquid nitrogen), survival increased when the cooling rate was increased to ~ 600 °C min^− 1^ using solid surface freezing of droplets (38 ± 7% recovery, 30 ± 5% hemolysis). When solid surface freezing was done with 400 mM trehalose, cell recovery was found to be 75 ± 18% with a concomitant hemolysis rate of 10 ± 2%, whereas trehalose loading was relatively low (12 ± 3 mM intracellular concentration). Similar findings were done with sucrose instead of trehalose in the freezing medium (data not shown).

### Cryoprotective properties of trehalose and betaine, and possible synergistic effects

**Fig. 2:**
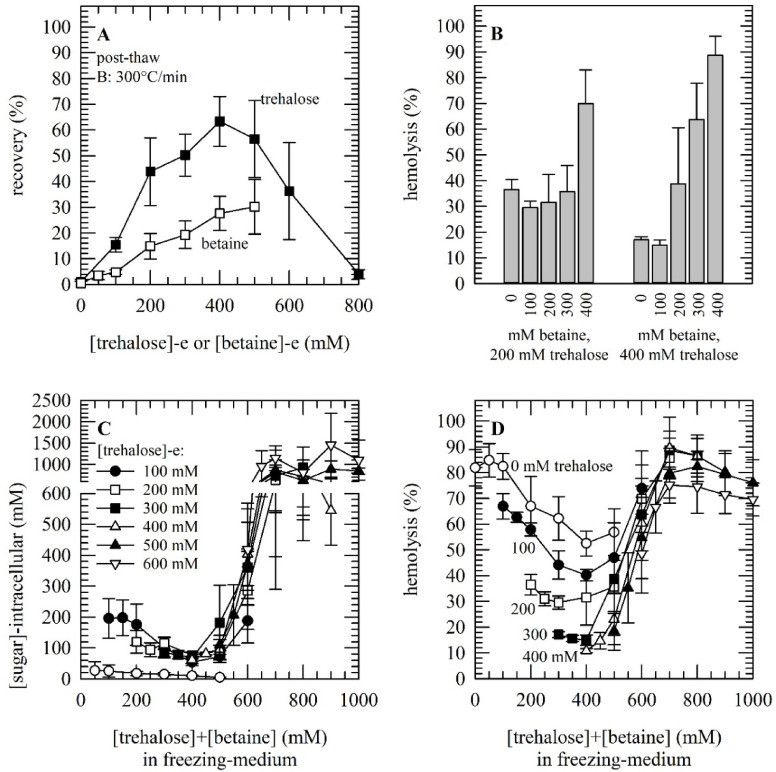
Recovery **(A)**, hemolysis **(B**,** D)** and intracellular sugar contents **(C)** of red blood cells after cryopreservation with different extracellular trehalose and betaine concentrations. RBCs were frozen using a cooling rate of ~ 300 °C min^− 1^ in 1×PBS with added up to 800 mM trehalose or 600 mM betaine alone **(A)** or in combination **(B − D)**. Panel **B** presents post-thaw hemolysis for using either 100 or 400 mM trehalose, combined with up to 400 mM betaine, whereas in panel **D** hemolysis is plotted versus the sum of the molarities of trehalose and betaine (i.e., plots presenting different extracellular trehalose concentrations; open circles: 0, closed circles: 100, open squares: 200, closed squares: 300, open upward triangles: 400, closed upward triangles: 500, downward open triangles: 600 mM). Also, post-thaw intracellular sugar concentrations are plotted versus the trehalose-plus-betaine content, i.e., for visualizing the effect of the external osmotic strength more clearly. Average ± standard deviation values are presented, determined using RBC samples originating from five **(A)** and three **(B − D)** different donors.

Next, cryoprotective properties of betaine were tested, when used alone (Fig. 2A) as well as in combination with trehalose (Fig. 2B − D). With fast cooling rates of ~ 300 °C min^− 1^, cryosurvival increased with increasing amounts of betaine, with recovering up to 30 ± 11% RBCs when using 500 mM betaine. This was lower, however, compared to that when using 500 mM trehalose as protective agent (57 ± 15%). The highest cell recovery was observed using freezing media containing 400 mM trehalose, namely 63 ± 10% (Fig. 2A) coinciding with a minimum in hemolysis (data not shown). Addition of betaine to 400 mM trehalose did not appear beneficial, with hemolysis actually increasing with adding more betaine. Adding betaine to samples containing less than 400 mM trehalose, however, had a positive effect, i.e., resulted in decreased hemolysis post-thaw (Fig. 2B). Interestingly, it appeared that hemolysis displayed a minimum when the sum of the amount of trehalose and betaine was 400 mM (Fig. 2D). In all cases, hemolysis rates were higher with trehalose-betaine combinations than with 400 mM trehalose alone (11 ± 1%).

Inspection of intracellular sugar concentrations post-thaw, indicated that loading efficiencies, i.e., [trehalose]_intracellular_ divided by [trehalose]_extracellular_, are predominantly dependent on the osmotic strength of the freezing medium. More specifically, the total amount of trehalose and betaine (i.e., amount of moles added and the medium osmolality) appears to be the major driving force and not the extracellular trehalose concentration (Fig. 2C). Use of freezing media containing 400 mM trehalose/betaine resulted in intracellular sugar concentrations ranging from 54 − 76 mM. Exceeding 500 mM concentrations resulted in higher intracellular sugar contents, however, at the expense of high hemolysis levels (> 75% with 600 mM trehalose/betaine). We note that loading efficiencies sometimes exceed 100%; particularly for fragile samples with high hemolysis levels. In this case, washing via centrifugation, which is needed to remove extracellular trehalose, causes cell losses and therewith inaccuracies in the determined intracellular trehalose contents.

### Use of betaine for limiting losses during washing of cryopreserved trehalose-loaded cells

**Fig. 3:**
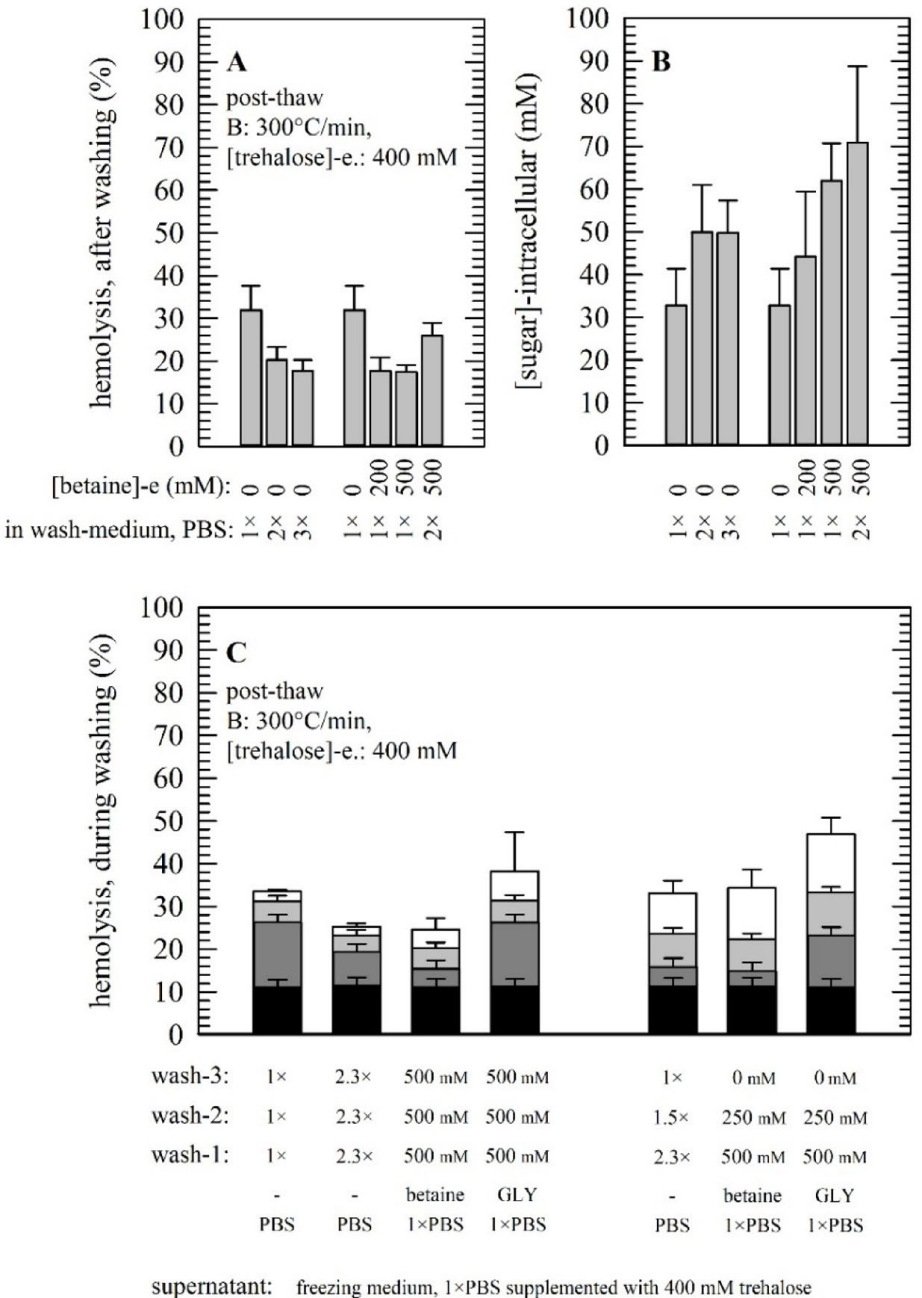
Effect of using different wash-media, on post-thaw outcomes of cryopreserved red blood cells which were loaded with intracellular trehalose. Loading was done through freezing at ~ 300 °C min^− 1^ in the presence of 400 mM extracellular trehalose. After thawing, RBCs were washed three times with 1 − 3×PBS, without supplements or supplemented with 200 or 500 mM betaine **(A**,** B)**; where after hemolysis **(A)** as well as intracellular sugar contents **(B)** were determined. Furthermore, the effect of using wash media supplemented with 500 mM betaine versus glycerol was tested, while gradually decreasing the concentration during washing or not **(C)**. Here, hemolysis was determined in the supernatant obtained directly after thawing as well as during three consecutive washing steps (presented as cumulative bars), i.e., while keeping the osmotic strength or gradually decreasing to isotonic conditions (1×PBS, 300 mOsm kg^− 1^). Average ± standard deviation values are presented, determined using RBC samples originating from five **(A**,** B)** and six **(C)** different donors.

Freezing-induced trehalose uptake increases the intracellular osmolality (> 300 mOsm kg H_2_O^− 1^) and hence post-thaw dilution of trehalose-loaded RBCs in isotonic medium (1×PBS) causes an osmotic shock and associated cell and/or hemoglobin losses. This is evident from the higher hemolysis found as compared to specimens washed and maintained in hypertonic saline (3×PBS) instead, namely 32 ± 6% vs. 18 ± 3% (Fig. 3A). In addition, we found that high intracellular trehalose concentrations were maintained when osmotic losses were prevented during washing, i.e., concentrations were 33 ± 9 and 50 ± 7 mM in 1× and 3×PBS, respectively (Fig. 3B). Betaine addition seems beneficial for counteracting losses during washing of cryopreserved trehalose-loaded cells. When using 1×PBS supplemented with 500 mM betaine, hemolysis was only 18 ± 3% and the intracellular trehalose concentration was maintained at 62 ± 9 mM (Fig. 3B). Hemolysis was found to increase with increasing betaine concentration and also when 500 mM betaine was added to 2×PBS.

In Fig. 3C it can be seen that gradually decreasing the betaine concentration during washing post-thaw (from 500 to 0 mM) resulted in hemolysis, eventually reaching similar levels as found for samples that were directly diluted in 1×PBS (followed by washing with fresh medium, three times), i.e., 32 − 34% hemolysis. Also, gradually decreasing the PBS strength from 2.3× to 1× during three subsequent washing steps could not reduce hemolysis and hemolysis levels were found to be the same as those directly diluted in 1×PBS. In case of direct dilution in 1×PBS, most of the hemolysis occurred during the first washing step, whereas hemolysis was distributed evenly among the subsequent washing steps upon gradual return to 1×PBS. Hemolysis occurring in subsequent wash steps with hypertonic media (2.3×PBS, 1×PBS supplemented with 500 mM betaine) can be attributed to fragile cells not surviving the osmotic stress and centrifugation procedure. This indicates that betaine does not rapidly permeate RBC membranes compensating for osmotic imbalances as we originally assumed. Therefore, we tested glycerol as a membrane permeating agent in combination with trehalose to see if it can reduce hemolysis, particularly during return to isotonic conditions after thawing. Addition of 500 mM glycerol to the wash medium resulted in high hemolysis values both with direct and gradual return to isotonic conditions.

### Membrane permeability of red blood cells, with special emphasis on the osmolyte betaine

**Fig. 4:**
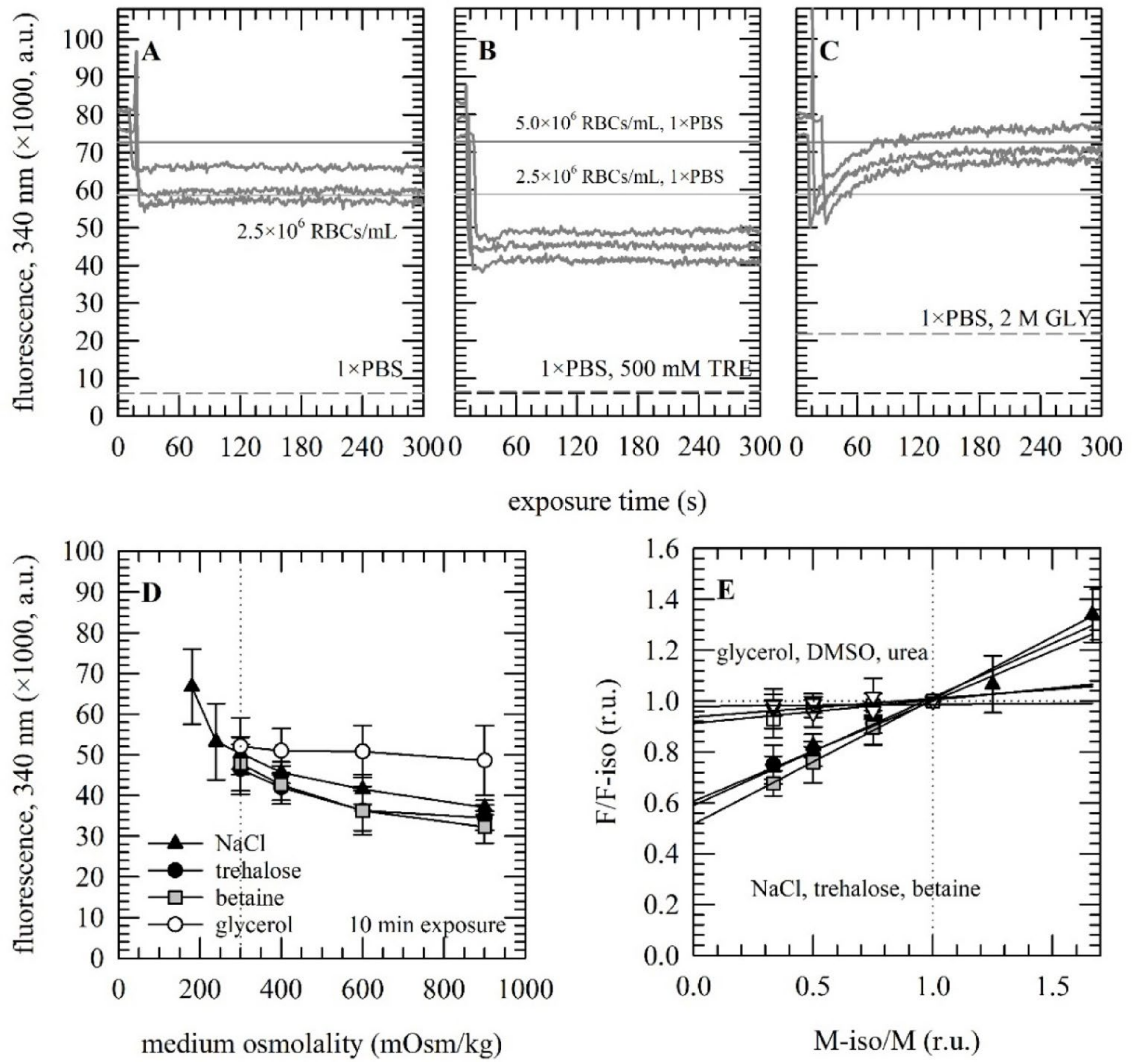
Osmotic responses of red blood cells diluted in PBS supplemented with various solutes, i.e., non-permeating and permeating cryoprotective agents. Autofluorescence at 340 nm was taken as a measure for the cell volume, and recorded versus time **(A − C)** as well as after 10 min exposure **(D**,** E)**. In panels **A − C**, besides traces collected for RBCs in the indicated media (three different donors), lines are shown representing undiluted and diluted cell samples in isotonic medium (5 × 10^6^ and 2.5 × 10^6^ RBCs mL^− 1^ in 1×PBS) as well as fluorescence readings obtained for solutions without addition of cells (**A**: 1×PBS without supplements; **B**: supplemented with trehalose; **C**: with glycerol). Panel **D** presents fluorescence values determined after equilibration versus the medium osmolality, i.e., in case of exposure to non-permeating (black symbols; triangles: NaCl, circles: trehalose) and permeating agents (white symbols; circles: glycerol, squares: DMSO, downward triangles: urea), as well as betaine (grey squares). In addition, the relative fluorescence (F/Fiso) is plotted versus the inverse relative osmolality (Miso/M) **(E)**. Average ± standard deviation values are presented, determined using RBC samples originating from 6 − 8 different donors.

The above cryosurvival and washing studies prompted us to investigate the membrane permeability of RBCs towards betaine. This was done by recording the autofluorescence of RBCs at 340 nm, i.e., upon dilution and incubation in media supplemented with additives used for cryopreservation and washing as described above. In Fig. 4A − C, it can be seen that RBCs diluted in isotonic medium exhibit a constant fluorescence emission, dependent on the cell concentration (see traces for 5 × 10^6^ and 2.5 × 10^6^ RBCs mL^− 1^ in 1×PBS). Dilution of RBCs in isotonic buffer results in a decrease in fluorescence intensity, which is simply due to a decrease cell concentration. Dilution in medium supplemented with trehalose results in a greater drop in fluorescence compared to dilution under isotonic conditions. This additional decrease in fluorescence (i.e., compared to dilution alone) can be attributed to fluorescence quenching caused by cell shrinkage. When RBCs are diluted in medium containing glycerol an abrupt decrease in fluorescence is observed followed by a gradual increase back to similar levels compared to cells kept in isotonic medium within 5 min. This reflects a typical shrink-swell volume response of cells that are exposed to a solution containing high concentrations of a membrane permeating solute. The slightly higher values after equilibration with glycerol are explained by background contributions (see trace of medium supplemented with glycerol only). We note that the initial data points (~ 15 s) are not reliable due to mixing effects and dilution of the sample. Test solution was directly added to RBC solution in the fluorometer followed by rapid mixing, which transiently disrupts the fluorescence readings. The increase in fluorescence at beginning of the measurement is due to opening the device.

In order to investigate the volume response of RBCs exposed to various membrane permeable and impermeable compounds as well as hypotonic conditions, fluorescence readings after 10 min exposure were plotted versus the medium osmolality (Fig. 4D). Exposure to hypotonic media results in an increase in fluorescence, whereas exposure to non-permeating solutes like sodium chloride and trehalose causes a concentration dependent decrease in fluorescence due to cell shrinkage. Exposure to betaine also results in a dose-dependent decrease in fluorescence indicating that betaine is membrane impermeable. In contrast, exposure to glycerol does not alter fluorescence values indicating it permeates into the cells.

A plot of the relative fluorescence (F/Fiso) versus the inverse relative osmolality (M/Miso) distinguishes membrane-permeable from membrane-impermeable solutes (Fig. 4E). Cells behave as so-called linear osmometers when exposed to membrane impermeable solutes, i.e., data can be fitted using a linear regression line. Cells only behave as ideal linear osmometers in a defined osmolality range. In severely hypertonic or hypotonic conditions, cells do not respond linearly to changes in medium osmolality. The relative fluorescence values correlate with the relative water content of the cell and the intercept on the y-axis reflects the residual osmotically insensitive fluorescence^32^. In contrast with this, in the presence of glycerol, DMSO, and urea normalized fluorescence values do not alter (i.e., remain ~ 1) with increasing osmolality.

### Cryopreservation of red blood cells using trehalose combined with permeating agents

**Fig. 5:**
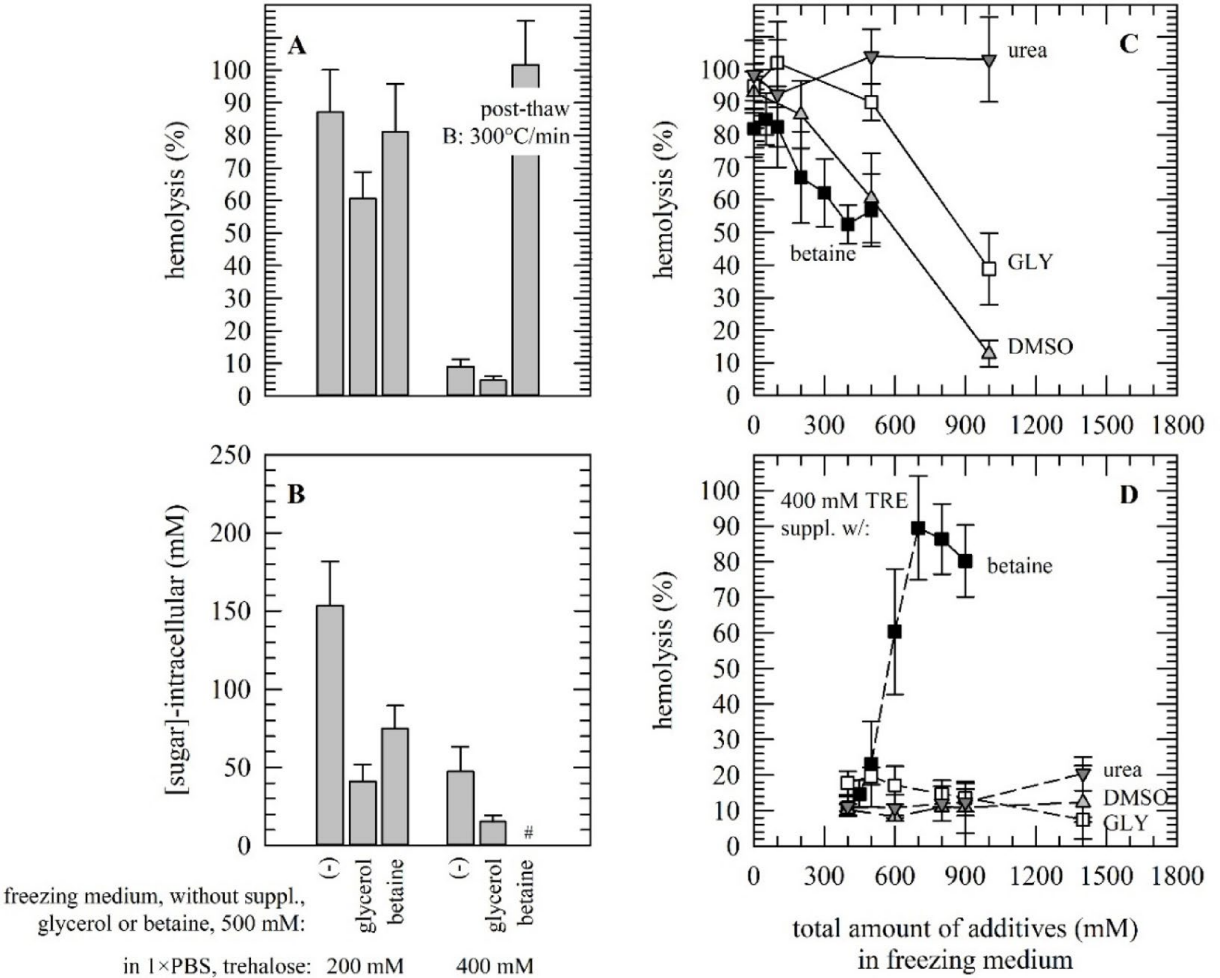
Effect of adding permeating agents to freezing media containing trehalose. RBCs were frozen at ~ 300 °C min^− 1^ in 1×PBS containing 200 or 400 mM trehalose, with and without 500 mM glycerol or betaine **(A**,** B)**, or with varying concentrations of permeating agents without and with 400 mM trehalose added **(C**,** D)**. Panel **A** and **B** show, respectively, post-thaw hemolysis and intracellular sugar contents (#: not determined). In panels **C** and **D**, post-thaw hemolysis data are plotted versus the total amount of additives added (in mM; black squares: betaine, white squares: glycerol, upward triangles: DMSO, downward triangles: urea); i.e., compounds alone (**C**; solid lines) or in combination with trehalose (**D**; dashed lines). Average ± standard deviation values are presented, determined using RBC samples originating from six **(A**,** B)** or three **(C**,** B)** different donors.

The effect of adding glycerol, DMSO, and urea to trehalose based freezing media on post-thaw hemolysis was compared with that of betaine. Figure 5A shows that adding 500 mM glycerol to freezing media containing 200 and 400 mM trehalose reduced post-thaw hemolysis. For the latter, hemolysis remained below 10%. In contrast, 500 mM betaine combined with 400 mM trehalose resulted in complete hemolysis, and almost complete hemolysis when combined with 200 mM trehalose (also see Fig. 2). Furthermore, it was found that intracellular sugar concentrations post-thaw decreased when glycerol was added; namely from 47 ± 16 to 15 ± 4 mM with using 400 mM extracellular trehalose (Fig. 5B).

Hemolysis remained low (< 20%) when adding up to 1000 mM glycerol to freezing media containing 400 mM trehalose (Fig. 5D). Similar low hemolysis levels were found for adding DMSO and urea, but not with betaine. Despite the fact that urea is membrane permeable, it does not have cryoprotective properties (Fig. 5C).

## Discussion

**Fig. 6:**
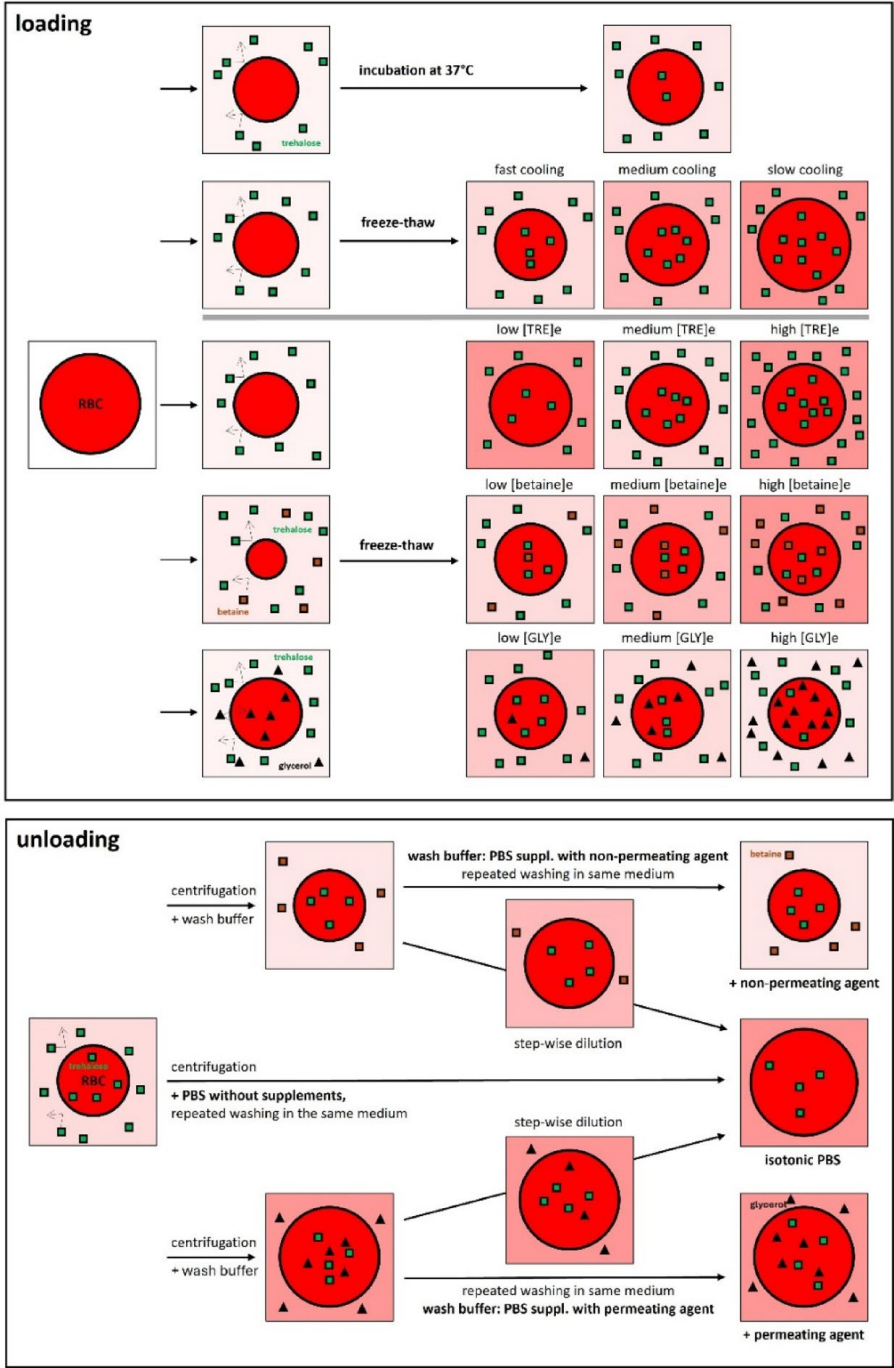
Schematic illustration of the osmotic movement of trehalose (small green squares) and concomitant hemolysis of RBCs during incubation, exposure to freezing-thawing in the presence of trehalose, and during washing and removal of extracellular trehalose. The effect of the cooling rate on freezing-induced trehalose uptake is illustrated for solutions containing trehalose alone and for trehalose in combination with permeable and non-permeating agents. Washing cryopreserved RBCs and trehalose unloading is shown for RBCs transferred in; hypertonic solutions containing betaine, isotonic PBS and PBS supplemented with glycerol (black triangles). In addition, solute transport processes are illustrated for frozen RBCs exposed to serial dilution from hypertonic wash buffer or PBS supplemented with glycerol to isotonic PBS. Permeating agents are displayed as triangles, non-permeating agents as squares.

When trehalose is used for cryopreservation of RBCs, this results in osmotic imbalances during the entire process; i.e., during loading, freezing, and return to isotonic conditions after thawing. This is schematically presented in Fig. 6. Trehalose uptake by RBCs during incubation at 37 °C increases in a time and concentration dependent manner at the expense of hemolysis. Exposing RBC/trehalose solutions to freezing-and-thawing results in relatively high intracellular trehalose concentrations, while uptake increases with exposure to higher extracellular trehalose concentrations and lowering the cooling rate. However, fast cooling rates are needed to obtain satisfactory post-thaw cell recovery and hemolysis. When using trehalose as sole CPA, and cooling rates of 300 °C min^− 1^ (i.e., plunging 1-mL samples in liquid nitrogen), optimal cryosurvival is found with freezing solutions containing 400 mM trehalose. Interestingly, when combining trehalose and betaine, cryosurvival remained optimal at a total solute concentration of 400 mM. Betaine also has cryoprotective properties but appeared less effective compared to trehalose. Whereas supplementing a 400 mM trehalose based freezing solution with betaine results in a rapid decline in cryosurvival, supplementation with membrane permeating agents like glycerol further increases cryosurvival. We assumed that betaine could act as a membrane permeating agent to alleviate hypertonic and hypotonic stresses associated with cryopreservation of red blood cells with trehalose as previously suggested^21^, but membrane permeability studies with RBCs clearly showed that betaine acts as a non-permeating agent. Directly returning trehalose-loaded cryopreserved cells to isotonic conditions after thawing results in hemolysis, which can be counteracted to certain extent using hypertonic solutions for washing.

Loading RBCs with trehalose by incubation at 37 °C is relatively inefficient, particularly compared to other cell types that possess endocytic pathways^20,33,34^. Trehalose uptake and hemolysis are the result of prolonged exposure of the cells to hypertonic conditions causing disruption of the membrane lipid bilayer, which can lead to altered fluidity and increased permeability. In contrast, we observed that simply subjecting RBCs to a freeze–thaw cycle in medium supplemented with trehalose strikingly enhances trehalose uptake. This finding is consistent with earlier reports for RBCs^29^ and other cell types^19^, and has been linked to freezing-induced fluid-to-gel membrane phase transitions, which transiently disrupt the membrane barrier function^9^. Trehalose uptake during freezing increases at lower cooling rates, likely due to more pronounced cellular and membrane dehydration under these conditions. Furthermore, uptake is driven by the osmotic gradient^19^, explaining the reduced loading efficiencies observed at higher cooling rates and when freezing solutions contain both trehalose and glycerol rather than trehalose alone. It is likely to assume that betaine also passes RBC membranes upon exposure to freezing-and-thawing. We note that trehalose uptake (and unloading) is inherently associated with damage, and there is a trade-off between achieving sufficient intracellular trehalose for protection and minimizing hemolysis.

Several studies have reported that cryosurvival can be enhanced by supplementing trehalose-based freezing solutions with natural, membrane-permeable osmolytes^23,25,35,36^. However, when we evaluated combinations of trehalose and betaine for RBC cryopreservation, no synergistic protective effect was observed. Notably, maximal cryosurvival occurred at a total solute concentration of 400 mM, irrespective of the relative proportions of trehalose and betaine. The decline in cryosurvival at concentrations exceeding 400 mM likely reflects the osmotic tolerance limit of RBCs (corresponding to a medium osmolality of approximately 700 mOsm kg⁻¹) beyond which cells become susceptible to solution effects injury^28^.

It has been proposed that betaine protects RBCs during cryopreservation by rapidly crossing the membrane via specific transporters^19^. However, our data indicate that betaine is not capable to rapidly pass through the RBC cell membrane. To assess membrane permeability, we monitored RBC autofluorescence as an indicator of cell volume changes following exposure to permeating and non-permeating solutes, as previously described^37^. A plot of the relative fluorescence versus the relative reciprocal osmolality showed a linear relationship for betaine, with an extrapolated osmotically insensitive fluorescence comparable to that observed for NaCl and trehalose, which indicates low permeability to betaine over a 10-minute exposure period. Using the same method, we confirmed the relatively rapid permeation of glycerol, DMSO, and urea, as RBCs exposed to these solutes returned to their initial fluorescence within minutes. For RBCs there is a strong positive correlation between relative cell volume and relative autofluorescence intensity^37^. Plotting relative fluorescence against the inverse relative osmolality yields a relationship analogous to a Boyle-van ’t Hoff plot, where V/Viso is plotted as a function of Miso/M.

Unlike betaine, supplementation of freezing solutions containing 400 mM trehalose with glycerol or DMSO enhanced RBC cryosurvival. A similar effect was observed with urea, which by itself lacks cryoprotective properties. These findings suggest that the synergistic protection provided by trehalose in combination with membrane-permeable solutes can be attributed to their rapid movement across membranes during osmotic stress.

Returning cryopreserved, trehalose-loaded cells to isotonic conditions after thawing is challenging because intracellular osmolality remains elevated relative to the extracellular environment. Intracellular trehalose causes the intracellular osmolality to exceed 300 mOsm, and hence exposing cells to isotonic buffer of 300 mOsm results in an osmotic imbalance and damage (hemolysis). In this study, we demonstrate that washing RBCs in hypertonic PBS supplemented with betaine (or other non-permeating solutes) reduces post-thaw hemolysis. Further optimization of washing procedures is needed; for example, stepwise re-equilibration to isotonic conditions through serial dilution may help minimize hemolysis and reduce post-thaw cell losses.

Taken together, the use of trehalose for cryopreservation of RBCs leads to osmotic imbalances throughout the entire process. Membrane-permeating agents may help mitigate these osmotic stresses. Betaine, however, did not appear capable of rapidly permeating RBC membranes. Although betaine demonstrated cryoprotective effects, it did not provide synergistic protection when combined with trehalose. In contrast, combining trehalose with membrane-permeating agents such as glycerol or DMSO, which can compensate for freezing-induced osmotic imbalances, shows promise for improving RBC cryopreservation outcomes.

## Material and methods

### Red blood cells, material and processing

Human blood was obtained from healthy volunteers with informed consent. Use of human blood was carried out after ethical approval according to legal provisions and rules of the Hannover Medical School (Ethics Committee of Hannover Medical School). Human blood samples in collection tubes with sodium citrate as anticoagulant (S-Monovette CPDA; Sarstedt, Nümbrecht, Germany), were provided by the Institute for Transfusion Medicine of the Hannover Medical School. These samples were aliquots originating from routine blood donations, prepared according to clinical standards, and their use for the studies described in this work was approved (MHH ethics committee, No.10163_BO_K_2022).

At the day of collection, whole blood samples (5 − 8 mL) were centrifuged (500×g, 5 min) and the supernatant and intermediate layer were removed. RBCs, recovered as pellet, were washed (500×g, 5 min, three times) and resuspended in phosphate buffered saline (1×PBS; Carl Roth, Karlsruhe, Germany). Cell concentrations were determined microscopically, using a Neubauer hemocytometer. RBC samples (1 − 5 × 10^9^ RBCs mL^− 1^) were stored at 4 °C for use within 7 days.

### Cryopreservation of red blood cells with different cooling rates

Cryopreservation of RBCs was primarily done using trehalose (Pfahnstiehl, Waukegan, IL, USA) as CPA, but also protective properties of betaine and typical permeating agents (glycerol, dimethyl sulfoxide/DMSO, urea) were tested. RBCs were prepared in 1×PBS (1 mL, 300 − 600 × 10^6^ RBCs mL^− 1^), and diluted with PBS and CPA stock solutions; to obtain samples containing 150 − 300 × 10^6^ RBCs mL^− 1^, in 1×PBS supplemented with 0 − 1000 mM trehalose, betaine, glycerol and/or DMSO.

One-mL samples in microtubes were frozen at different cooling rates, i.e., by directly plunging in liquid nitrogen (~ 300 °C min^− 1^) or by using a controlled rate freezer (IceCube 14 M; SY-Lab; Neupurkersdorf, Austria) with cooling rates set between 1 and 60 °C min^− 1^. Actual cooling rates were measured using a thermocouple inserted in a mock sample. Samples were stored in liquid nitrogen.

Thawing was done in a 37 °C water bath until no more ice was visible (~ 3 min). Hemolysis and cell recovery percentages were taken as measures for cryosurvival. Therefore, specimens were subjected to centrifugation (2000×g, 1 min), and analyses were done on the initial supernatant as well as during washing (three times). As washing medium, typically 1×PBS supplemented with 500 mM betaine was used, however, also iso- (1×) and hypertonic PBS (up to 3×), and 1×PBS supplemented with glycerol and different betaine concentrations were tested (i.e., while maintaining the medium osmolality constant or with stepwise decreasing to 1×PBS).

In addition to cryopreservation of 1 mL samples in cryovials, solid surface freezing of droplets (20 µL, 2 × 10^9^ RBCs mL^− 1^) was pursued to achieve faster cooling rates (~ 600 °C min^− 1^). This was done as described in detail elsewhere^38^, using a liquid-nitrogen-cooled copper block for freezing. Thawing was done by transferring 5 frozen droplets in 900 µL warm (37 °C) 1×PBS supplemented with 500 mM betaine (resulting in 200 × 10^6^ RBCs mL^− 1^).

### Determination of hemolysis and recovery of red blood cells

Hemolysis was determined from recovered supernatants after centrifugation (2000×g, 1 min) by measuring the absorbance at 541, 576 and 630 nm using a Synergy MX microplate reader (BioTek by Agilent; Santa Clara, CA, USA). Using these wavelengths, heme contents and the degree of oxidation was determined^39^. Hemolysis was calculated by dividing sample absorbance values by those determined after complete hemolysis, i.e., after adding 0.05% (v/v) Triton X-100 to the sample. Cumulative hemolysis was calculated by adding hemolysis values, i.e., for the supernatant and subsequent washing steps. Cell recovery percentages were determined by dividing RBC numbers originally present in the sample by those recovered after a specific treatment.

### Assessment of intracytoplasmic sugar contents

The anthrone assay was used to determine intracellular trehalose contents (Dreywood 1946). This was done for RBCs suspended in 1×PBS supplemented with trehalose (0 − 1000 mM); after incubation at 37 °C as well as after subjecting to freezing-and-thawing. RBCs were washed three times by centrifugation (1 min at 2000×g). After washing, cell numbers were determined and cells were collected in a pellet. To extract sugars, cell pellets were suspended in 800 µL 80% methanol and incubated at 80 °C for 45 min (while shaking). Samples were centrifuged (200×g, 10 min, ~ 22 °C), to remove cellular remnants in the pellet, while the supernatant was transferred into a new tube for drying at 55 °C. Dried samples were dissolved in 1 mL water, from which an aliquot of 75 µL was taken and mixed with 300 µL anthrone reagent (2 mg mL^− 1^ anthrone, in 99% sulfuric acid). Samples were incubated for 10 min at 99 °C (while shaking). After cooling, the absorption at 620 nm was measured, using the microplate reader. Values were compared with a standard curve prepared using pure trehalose. The cytoplasmic trehalose concentration was calculated by dividing the amount of trehalose in moles by the osmotically active cell volume of the cell pellet^40^, assuming a mean cell volume of 91 µm^3^ and a cytoplasmic volume of 53 µm^3^. The loading efficiency was calculated as the ratio between the cytoplasmic and the extracellular trehalose concentration.

### Assessment of osmotic responses to different solutes

Osmotic responses of RBCs towards various cryoprotectants and osmolytes were evaluated as previously described^37^. This method relies on measuring the autofluorescence of endogenous proteins in RBCs (predominantly hemoglobin), via the fluorescent emission of tryptophan. Autofluorescence was measured using excitation and emission wavelengths set at 280 ± 10 and 340 ± 5 nm, respectively, using a fluorescence spectrophotometer (FL8500; PerkinElmer, Waltham, MA, USA). Fluorescence emission of RBCs is proportional to cell volume; emission intensity decreases during cell shrinkage due to fluorescence quenching and likewise increases during cell swelling.

Osmotic responses were tested towards typical additives used for cryopreservation and washing (sodium chloride, trehalose, betaine, glycerol, DMSO and urea). Concentrated solutions of these compounds were prepared (1 − 5 M, in water) and diluted with 5×PBS and water to prepare solutions in 1×PBS at two-fold the desired final concentration (0 − 900 mM). Two-fold solute solution was mixed with an equal volume of RBC sample (5 × 10^6^ cells mL^− 1^, in 1×PBS); resulting in 2.5 × 10^6^ cells mL^− 1^, 1×PBS, and osmolalities ranging from 300 − 1200 mOsm kg H_2_O^− 1^. Samples were incubated for 10 min at room temperature, followed by gentle mixing and fluorescence measurements. For selected conditions, RBC volume responses were monitored over time by taking fluorescence readings at 1 s time intervals.

Fluorescence values were plotted versus time or the medium osmolality. In addition, data are presented in plots; in which the fluorescence (F) in medium at a given osmolality (M) is divided by the value determined in isotonic medium (Fiso, at Miso of 300 mOsm kg H_2_O^− 1^) and plotted versus the reciprocal of the normalized osmolality (Miso/M). In case viable cells are exposed to membrane impermeable solutes, the intercept on the y-axis in a plot of F/Fiso versus Miso/M reflects the residual osmotically inactive fluorescence^32^.

## Data Availability

Research data generated in this study are available upon reasonable request.
